# Management of Adenovirus in Children after Allogeneic Hematopoietic Stem Cell Transplantation

**DOI:** 10.1155/2013/176418

**Published:** 2013-10-28

**Authors:** Winnie WY Ip, Waseem Qasim

**Affiliations:** ^1^Molecular Immunology Unit, UCL Institute of Child Health, 30 Guildford Street, London WC1N 1EH, UK; ^2^Department of Clinical Immunology, Great Ormond Street Hospital, London WC1N 3JH, UK

## Abstract

Adenovirus (ADV) can cause significant morbidity and mortality in children following haematopoietic stem cell transplantation (HSCT), with an incidence of up to 27% and notable associated morbidity and mortality. T-cell depleted grafts and severe lymphopenia are major risk factors for the development of adenovirus disease after HSCT. Current antiviral treatments are at best virostatic and may have significant side effects. Adoptive transfer of donor-derived virus-specific T cells has been shown to be an effective strategy for the prevention and treatment of ADV infection after HSCT. Here we review progress in the field and present a pathway for the management of adenovirus in the posttransplant setting.

## 1. Introduction

Adenovirus (ADV) causes mild illnesses in immunocompetent hosts but can cause significant morbidity and mortality in the immunocompromised, for example, children in the posthaematopoietic stem cell transplant setting. Haematopoietic stem cell transplantation (HSCT) can offer a cure for many haematological diseases, primary immunodeficiencies, and inborn errors of metabolism. However, not all transplant recipients have fully matched sibling donors and alternative donor sources have to be sought. In HLA-matched or mismatched unrelated donor setting, conditioning regimens will often include serotherapy such as Alemtuzumab (monoclonal anti-CD52 antibody) or thymoglobulin (polyclonal horse or rabbit thymocyte globulin [ATG]) to remove alloreactive T cells in the recipient that can cause acute Graft versus Host Disease (GVHD). During the posttransplant period of reduced T-cell immunity when reconstitution of donor-derived immune system is slow and the use of immunosuppressive agents is necessary, transplant recipients are especially vulnerable to viral reactivations and/or infections. 

Whilst antivirals such as ribavirin and cidofovir are available for the treatment of ADV, they are associated with toxicity and have variable efficacy. Over the past decade or so, adoptive transfer of donor-derived virus-specific T cells has been explored extensively as an alternative method to prevent and treat ADV and other viral infections after HSCT. This review examines recent preclinical and clinical studies on T-cell immunotherapy for ADV and provides a strategy for monitoring and management of ADV in children after allo-HSCT. 

## 2. Adenovirus

**Figure 1:**
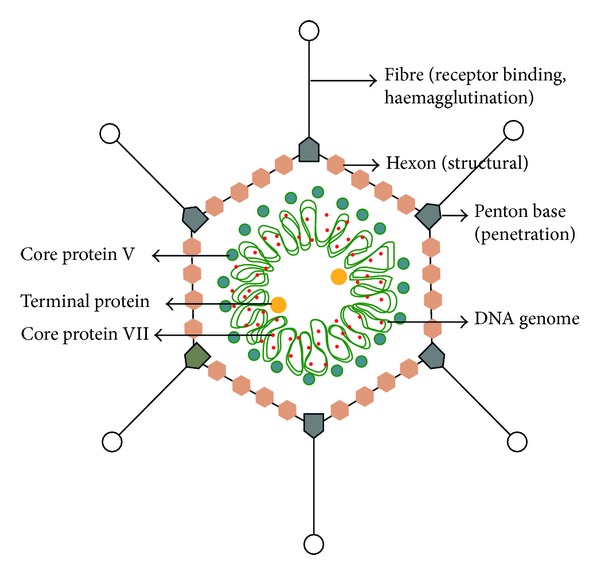
Structure of adenovirus.

Adenoviruses (ADV) were first isolated in 1953 from human adenoid tissues obtained during adenoidectomy [1]. They are nonenveloped, double stranded DNA viruses that range in size from 65 to 80 nm in diameter [2]. To date, over 60 ADV types have been identified, which can be classified into seven subgroups, A–G, on the basis of their haemagglutination properties, their oncogenic potential in rodents and DNA homology, or GC content of their DNA (Table 1) [3–6]. The virion is composed of 252 capsomers: 240 hexons and 12 pentons arranged in an icosahedral shape and a nucleoprotein core that contains the DNA viral genome and internal proteins. The linear, double stranded DNA genome is 34–36 kb in size and encodes for more than 30 structural and nonstructural proteins [5, 6]. Each penton in the capsid comprises a base and a rod-like outward fibre projection of variable length depending on serotypes [2, 7]. The hexon contains group-specific antigenic determinants in addition to subgroup-specific determinants and type-specific neutralising epitopes (Figure 1) [7]. Tissue tropism of ADV differs among the different serotypes but generally corresponds to the subgroups. Subgroups C and E and some B viruses typically infect the respiratory tract; other B serotypes infect the urinary tract (B11, B34, and B35); serotypes from subgroups A and F target the gastrointestinal tract and serotypes from species D the eyes [5, 8]. 

## 3. Humoral Immunity Against ADV

Hexon, fibre, and to a lesser extent penton have been shown to be the major targets for ADV neutralising antibodies (Nab) [9–12]. After HSCT, subjects who developed ADV viraemia but subsequently cleared the infection have detectable humoral immune responses after a period of several weeks to months after viral clearance, with titres of serotype-specific Nabs increasing by 8–16-fold. Interestingly in some cases, preexisting, high titres of ADV-specific Nabs in serum did not prevent progression to viraemia [13]. Whilst humoral immunity clearly plays a critical role in anti-ADV immunity, administration of immunoglobulin therapy has not been shown to be effective in preventing ADV reactivation or of proven benefit for the management of established viraemia or organ specific infection [14, 15]. 

## 4. Cell-Mediated Immunity Against ADV

Cellular immunity towards adenoviruses has been extensively studied over the past two decades and has been found to be cross-reactive across serotypes, confirming the presence of conserved antigens [16–21]. In a group of 8 healthy subjects with no serologic evidence of prior exposure to the uncommon group B Ad35, specific CD4+ proliferation has been shown to both Ad2- and Ad35-infected cell lysates [16]. In humans the response is predominantly in CD4+ T cells specific for capsid derived antigens [16, 19, 20], but cytotoxic responses are also found in CD8+ T cells against both viral structural and recognition proteins. The immunodominant CD4+ and CD8+ T-cell epitopes are found located in the major capsid protein hexon and have been found to induce T cells that are either broadly cross-reactive or reactive within particular subgroups [20–24]. Healthy adults have low frequencies of ADV hexon-specific CD8+ (38%) and CD4+ (81%) T cells detected in peripheral blood [25]. In a group of 8 healthy subjects with no serologic evidence of prior exposure to the uncommon group B Ad35, specific CD4+ proliferation has been shown to both Ad2- and Ad35-infected cell lysates [16]. 

## 5. Adenoviral Infection in the Immunodeficient Host

Adenovirus is endemic in paediatric populations with 80% of children between 1 and 5 years of age having antibody to one or more serotypes. Infections in immunocompetent hosts are usually benign and short-lived and most commonly manifest as upper respiratory tract infections [26]. ADV causes 2–7% of respiratory tract infections in children in the first 5 years of life and is responsible for 5–11% of cases of viral pneumonia and bronchiolitis in infants and children. Illness typically lasts less than 2 weeks, but once infected the virus remains latent in lymphoreticular tissue including tonsils, adenoids, and intestines. In healthy children viral shedding can persist for months or years. In the immunocompromised patients ADV can cause severe and protracted systemic illnesses such as hepatitis, pneumonitis, colitis, haemorrhagic cystitis, and encephalitis [26, 27]. The immunosuppressed paediatric host is particularly susceptible, most notably in the allogeneic transplant setting where cellular immunity is compromised. The incidence of ADV infection reported in bone marrow transplant recipients ranges from 5% to 29% in earlier studies where ADV was detected via routine weekly surveillance cultures up to first 100 days after transplant [7, 26, 28–32]. The advent of robust PCR based detection meant that serial ADV PCR has become the mainstay of routine surveillance, with incidence of viral isolation reported as between 17 and 27% in paediatric transplant recipients [4, 33]. 

These retrospective and prospective studies have facilitated the identification of several risk factors that are predictive for the development of ADV infection and/or disease in transplant recipients. One risk factor identified is T-cell depletion either *ex vivo* by CD34+ positive selection or *in vivo* with Alemtuzumab or antithymocyte globulin (ATG). In a group of 153 children receiving HSCT, adenoviraemia occurred in 26 children (17%), all of whom had received T-cell depleted grafts. Similarly Lion et al. found a significant increase in incidence of ADV infection in group of paediatric patients transplanted with T-cell depleted grafts [4]. And in a cohort of 76 adult allograft recipients ADV was isolated exclusively in recipients of Alemtuzumab mediated T-cell depleted grafts (15 of 76, 20%) [34]. 

Detection of ADV infection at multiple sites has also been correlated with increased risk for invasive disease in children [4, 30, 31]. However, the most significant predictor of adenovirus infection identified in the majority of studies was lymphopenia, with all patients who developed adenovirus disease or with persistent adenoviraemia having an absolute lymphocyte count (ALC) of less than 300/*μ*L [33, 34]. In patients with established adenoviraemia, an increase in lymphocyte counts correlated with clearance of infection and survival of the host whereas those who died of adenoviraemia had continuously increasing ADV DNA loads in plasma with no lymphocyte recovery [13]. To further illustrate the importance of immune reconstitution in clearance of adenoviraemia, 46 children after HSCT were prospectively studied. Children who died (7/21) of ADV infection had no adenovirus-specific T cells and had significantly reduced T-cell reconstitution, although absolute lymphocyte count was above 0.3 × 10^9^/L at 30 days after transplant. Ninety-three percent of patients who successfully cleared ADV infection had presence of virus-specific T cells, compared to 54% of children without any ADV infection. They also had good T-cell reconstitution, especially CD8+ T cells (>0.4 × 10^9^/L) at 60 days after transplant [35]. In the current era of prospective monitoring, Hiwarkar et al. considered the impact of ADV reactivation in 291 paediatric HSCT procedures and again found reduced CD4 counts of less than 0.15 × 10^9^/L in the first 3 months after transplantation as a significant risk factor for developing adenoviraemia [36]. The overall mortality from ADV infection after HSCT ranges from 6% and 60% in studies of mixed populations with adults and children [7, 29, 30, 32] and between 19% to 83% amongst paediatric patients [26, 33, 37].

## 6. Diagnosis and Monitoring 

The development of real-time quantitative PCR assays has allowed for accurate detection of ADV in a variety of tissues, including blood, stool, and urine [2] and allows for prospective monitoring of adenoviraemia in the posttransplantation setting. Kampmann et al. report a median of 21 days after transplant before adenoviraemia was evident [33]. In a prospective study that identified 21 paediatric HSCT patients with ADV infection, 90% of infections occurred during the first 3 months after transplant, with more than 50% of patients having ADV infection within 30 days after HSCT [35]. The most prevalent group of adenovirus identified had been subgroup C [4, 33, 34], and subtypes 2, 5, 1, 6, 31, and 4 (in decreasing frequency) are the most prevalent [38].

There is a correlation between high plasma viral load and fatal outcome or invasive disease, with those who died of disseminated ADV disease having a much higher ADV DNA load than patients who survived [39, 40]. There is also association between onset of ADV-related disease and mortality. In a study of 132 consecutive paediatric patients undergoing SCT, 91% of those who were ADV positive in peripheral blood died, with adenoviral DNA detected in blood at a median of 29 days before death. And in those who developed disseminated disease, virus was detected in blood by a median of more than 3 weeks before onset of clinical symptoms [4]. These earlier studies all suggest that high viral load precedes symptoms of disseminated disease; therefore prospective monitoring of ADV load is now implemented in many of the paediatric transplant centres.

## 7. Management of Adenoviral Infection in Immunocompromised Children

### 7.1. Antiviral Drugs

Cidofovir is an acyclic nucleoside phosphonate derivative of cytosine which is converted to an active intracellular metabolite, cidofovir diphosphate, by cellular kinases [14, 41]. The active intracellular diphosphate form of the drug exerts its mechanism of action as both a competitive inhibitor and an alternative substrate for 2′-deoxycytidine 5′-triphosphate in the viral DNA polymerase reaction [42], thus inhibiting viral replication. Antiviral selectivity results from the higher affinity for the viral DNA polymerase compared to cellular DNA polymerases [8]. 

Several studies have reported on the success of CDV in the treatment of ADV infection in immunocompromised hosts after HSCT [14, 42], especially when given early [43–46] and combined with withdrawal of immunosuppression [33]. At our centre, if blood ADV reaches >1000 copies per mL on two consecutive occasions CDV is started at 5 mg/kg once every week for 2 weeks, followed by maintenance dose of 5 mg/kg once every fortnight. Notable side effects include nephrotoxicity, especially when used in combination with other nephrotoxic drugs such as cyclosporine or tacrolimus [15]. CDV is a dianion that is taken up into the proximal renal tubular cells by an organic anion transporter at the antiluminal membrane. Once taken up into the cells, a slow diffusion rate into the tubule lumen, as well as CDV's long intracellular half-life, can lead to toxic intracellular accumulation and subsequent tubular necrosis. Toxicity can be reduced by concomitant use of oral probenecid and intravenous hyperhydration [41]. Probenecid competes for the kidney anion transporter and along with hyperhydration can help protect tubular cells by decreasing plasma clearance rate of CDV [47, 48]. Ljungman et al. published two studies each on the use of CDV as therapy for ADV and cytomegalovirus infection in 126 stem cell transplant patients combined [44]. The risk of renal toxicity in both studies was 26% and most of the renal toxicity was mild (low-degree proteinuria or mild elevation of serum creatinine), but approximately half had remaining signs of renal impairment after discontinuation of CDV [44].

Ribavirin is a nucleoside analogue for which *in vitro* anti-ADV activity has been reported but it differs against different subtypes. It is active on most ADV isolates from species A, B, and D and in all isolates from species C [49]. There is anecdotal evidence of successful treatment of ADV in immunocompromised patients but larger studies have not been as supportive [43, 50] (reviewed in [15]). There is no provable role for ganciclovir or for immunoglobulin therapy in immunocompromised patients [14, 15]. 

More recently a new oral therapy has been trialled for the treatment of ADV infections in immunocompromised patients. CMX001 (hexadecyloxypropyl cidofovir) is an orally bioavailable lipid conjugate of cidofovir with good oral bioavailability and can achieve higher intracellular levels of active drug compared with cidofovir; it may also have a better safety profile [51]. The drug was trialled in 13 immunocompromised patients and nearly two-thirds had a ≥10-fold drop in viral load after 1 week of therapy [51]. CMX001 is currently being studied in a randomised, placebo-controlled, Phase 2 trial for the preemptive treatment of adenovirus disease versus placebo in 48 paediatric and adult haematopoietic cell transplant recipients (ADV Halt Trial, NCT01241344). 

### 7.2. Withdrawal of Immunosuppression

**Figure 2:**
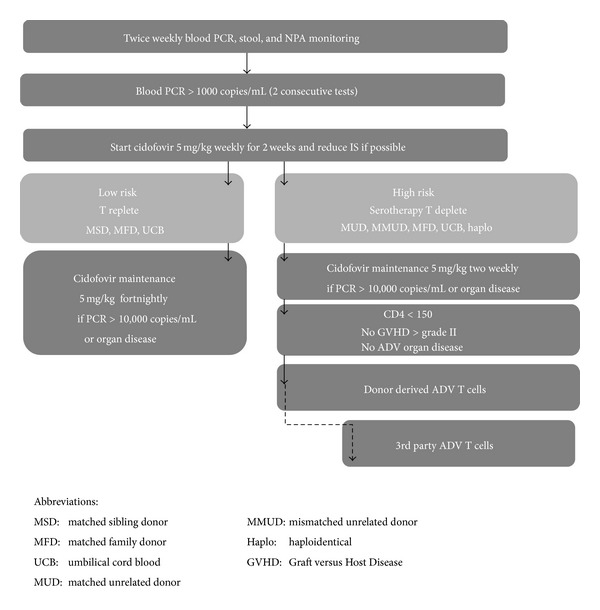
Algorithm for the management of ADV reactivation in children after allogeneic stem cell transplantation.

Chakrabarti et al. reported on the success of pre-emptive reduction or withdrawal of immunosuppressive therapy at first detection of adenovirus. In a group of 76 adult allograft recipients, 15 developed adenovirus disease/infection. Twelve patients had immunosuppression withdrawn or reduced and 9 had resolution of infection, whereas all 3 patients in whom immunosuppression had to be continued succumbed to adenovirus disease [34]. Similarly in paediatric transplant recipients withdrawal of immunosuppression together with early antiviral therapy led to the resolution of adenoviraemia in 19/26 (86%) patients [33]. Hence in the posttransplant setting we would recommend the following algorithm for the treatment of ADV (see Figure 2).

### 7.3. Adoptive Immunotherapy

It is clear that recovery from ADV infection requires cellular immune reconstitution after allogeneic HSCT. Adoptive immunotherapy using both unmanipulated T cells and virus-specific T cells has therefore been evaluated as approaches to reconstitute antiviral immunity. 

Unmanipulated donor lymphocyte infusions (DLI) containing virus-specific T cells have been trialled in patients with EBV infections which resulted in clearance of infection but increased the risk of GHVD due to high frequency of alloreactive cells [52]. In 1995 Walter et al. infused clones of CMV-specific CD8+ cytotoxic T lymphocytes from donors into 14 recipients of allogeneic bone marrow in an attempt to reconstitute cellular immunity against CMV. All 14 patients had reconstituted CMV immunity by days 42 to 49 after marrow transplantation. The transferred CD8+ clones persisted for at least 8 weeks after completion of T-cell therapy [53]. 

Hromas et al. reported the first successful treatment of adenovirus infection with DLI. The patient developed severe ADV-associated haemorrhagic cystitis after a T cell depleted graft and did not respond to antiviral drugs or immunoglobulin. After infusion of 1 × 10^6^/kg CD3+ cells on day +61 the patient improved over a period of 5 weeks without developing GVHD. This successful treatment supported the rationale for the adoptive transfer of adenovirus-specific CTL [54]. Earlier studies on adoptive immunotherapy have been summarised in recent reviews [47, 55–58]. 

## 8. Generation of T Cells Against ADV

In order to increase antiviral efficacy and to reduce the risk of alloreactivity, techniques were developed to isolate only ADV-specific T cells to be given to patients. Smith et al. in 1996 used donor peripheral blood dendritic cells as antigen-presenting cells to manufacture cytotoxic T cells (CTLs) that recognise ADV. Dendritic cells (DCs) from donors were infected with either wild-type adenovirus serotype 5 (Ad) or Ad5 strain *dl312*, an Ad5 mutant with the E1A region deleted resulting in a virus defective in early and late viral gene transcription. The adenovirus-specific T cells were subsequently expanded using virion-pulsed irradiated DCs [17]. The majority of the CTLs were CD4+ T cells and were directed against the input virion proteins. They demonstrated cross-reactivity but were unable to kill target cells in a standard 4–6-hour assay (requiring 18 hours to kill) and could not be adequately expanded into CTL lines [59]. 

Following on from this, in 2004 Leen and her group developed a protocol to reactivate ADV-specific memory T cells from donors' PBMCs using clinical-grade ADV vector. PBMCs from 6 healthy ADV-seropositive volunteers were stimulated with autologous dendritic cells (DCs) transduced with Ad5f35 (replication-defective ADV vector). CD4+ and CD8+ ADV-specific T cells were isolated and expanded with autologous EBV-transformed lymphoblastoid cell lines (LCLs) and showed ADV specific killing [59]. Because the generation of DCs to act as stimulator cells requires a large volume of blood, a second protocol using Ad5f35GFP-transduced PBMCs as both stimulators and responders was used. Expansion was again carried out with LCLs, and the resultant expanded T cells had specific reactivity against both ADV and EBV. These CTL lines generated using Ad5f35 vector were able to recognise and kill autologous cells infected with wild-type adenovirus isolates from different serotypes and groups including Ad2, Ad4, Ad7, and Ad11 [59].

**Figure 3:**
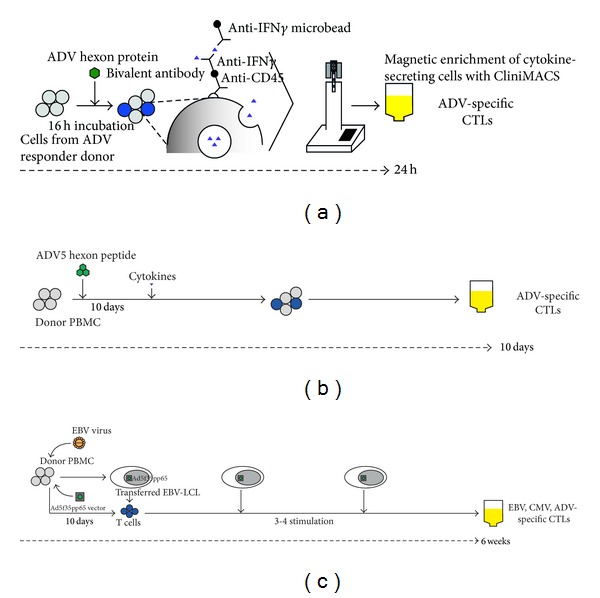
Protocols for generating virus-specific T cells. (a) Donor identified as ADV responder by IFN-*γ* secretion assay (Miltenyi Biotec, Bergisch Gladbach, Germany). Peripheral blood mononuclear cells (PBMC) isolated and incubated overnight with ADV Hexon protein. Responding cells captured with IFN-*γ* reagent, anti-IFN-*γ* microbead and magnetically enriched with CliniMACS (Miltenyi Biotec) [60]. (b) Donor PBMC incubated over 10 days with ADV5 hexon peptide and cytokines. Expanded cells isolated and infused into patients after QA/QC testing [61]. (c) EBV-transformed B cell lines (EBV-LCLs) generated from donor PBMCs by infecting with EBV virus. Donor PBMCs are transfected with Ad5f35pp65 vector (replication-competent adenovirus-negative) and later restimulated several times by EBV-LCLs that have been transduced with the same vector [62].

In 2006 Leen et al. reported on the prophylactic clinical use of trispecific (EBV, CMV, and ADV) CTLs on 11 adult and paediatric patients after haematopoietic stem cell transplant. Donor PBMCs transduced with a recombinant adenoviral vector encoding the CMV antigen pp65 (Ad5f35pp65) were used to reactivate CMV- and ADV-specific T cells. Subsequent stimulation with EBV-transformed LCLs transduced with the same vector reactivated EBV-specific T cells whilst maintaining the expansion of activated ADV- and CMV-specific T cells (Figure 3(c)). Fifteen donor CTL lines were generated and all showed cytolytic activity against all three viruses [62, 63]. Eleven patients received from 5 × 10^6^ to 1 × 10^8^ cells/m^2^ at 35 to 150 days after HSCT. CMV- and EBV-specific CTLs consistently expanded in all individuals treated within 4 weeks of administration, whereas ADV-specific CTLs expanded only in those with active or recent infection. All patients with preinfusion viral infection/reactivation had reduction in viral titre and resolution of disease symptoms, contemporaneous with expansion of virus-specific T cells detected in peripheral blood [62].

In order to increase the frequency of adenovirus-specific T cells within their CTL lines, Leen and her group removed competition from the immunodominant CMV antigen and manufactured bivirus-specific CTL lines directed only to EBV and adenovirus [64]. Twenty CTL lines were made, of which 13 were administered to paediatric stem cell transplant recipients: 7 unrelated and 6 haploidentical transplants. The frequency of adenovirus hexon-specific T cells in the bivirus CTL was significantly higher than in the trivirus CTL study (median ADV cells 308 spot forming colonies/10^5^ CTL [range 46–350] compared to 86 SFC/10^5^ CTL [46–350] in bivirus product) [62, 64]. Each patient received from 5 × 10^6^ to 1.35 × 10^8^ cells/m^2^ at 40 to 150 days after HSCT. There were no toxicities related to CTL therapy and no subject developed de novo GVHD after cell infusion. None of the 13 patients developed EBV-associated lymphoproliferative disease, and 2 of the subjects had resolution of their adenoviral disease [64]. More recently in a multicentre study, banked third-party virus-specific T cells (VSTs) were administered to 50 patients with severe, refractory CMV, ADV, or EBV infections [65]. Thirty-two virus-specific lines were generated from individuals with common HLA polymorphisms immune to EBV, CMV, or ADV, of which 18 lines were administered to 50 post-HSCT patients with severe, refractory illness due to infection with one of these viruses. The virus specific T cells were generated by transduction of PBMC with clinical-grade Ad5f35pp65 vector followed by stimulation with EBV-transformed LCL that had been transduced with the same chimeric vector. The VSTs were then cryopreserved until required. Patients were excluded from the study if they had received T-cell serotherapy within 28 days of proposed administration date. The cumulative rates of complete or partial responses at 6 weeks after infusion were 74% for the entire group. No immediate infusion-related adverse events were noted, 2 patients developed de novo GVHD (grade 1). Six out of eight patients who did not have line available and continued with standard therapy died of their viral disease. This approach of using “off-the-shelf” third-party VSTs is promising as it appears to remove some of the barriers to the wider application of cell therapy in viral reactivations posttransplant. It avoids the lengthy time and cost of producing individual lines, and does not appear to cause GVHD from alloreactivity of the third-party cells [65].

Once it has been established that ADV-specific T cells can be expanded *in vitro* and that they are effective and protective *in vivo, *the next challenge was to overcome logistics of manufacturing these products. The protocol described above of activating donor PBMCs with autologous monocytes transduced with the Ad5f3pp65 vector followed by restimulation with Ad5f35pp65-transduced EBV-LCL takes in total 10–14 weeks. This implies that products have to be manufactured in advance if they were to be made immediately available for acutely ill patients; and comes with it cost implications. Different cell selection and culture practices were therefore explored to develop more rapid and cost-effective strategies for production of CTLs.

## 9. Cytokine Based Selection of Antigen-Specific T Cells from Donor Peripheral Blood Mononuclear Cells

In 2004 Feuchtinger et al. described a clinical-grade strategy to isolate and expand donor derived human ADV-specific T lymphocytes using the Miltenyi Biotec (Bergisch Gladbach, Germany) interferon-*γ* (IFN-*γ*) secretion assay. PBMCs were isolated from suitable donors and stimulated with type C adenoviral antigen (BioWhittaker, Verviers, Belgium) for 16 hours. T cells with antigen-specific secretion of IFN-*γ* were detected on the following day and these cytokine-secreting cells were magnetically enriched using CliniMACS device (Miltenyi Biotech). A mean number of 3.4 × 10^6^ cells were obtained with a mean purity of 85% ADV-specific T cells. These isolated cells were then expanded *ex vivo* in a median of 18 days (range 7–29 days) to greater than 10^8^ total cells using IL-2 and autologous feeder cell stimulation. The generated T cells showed ADV-specific IFN-*γ* release and specific killing of ADV-infected cells. Alloreactive proliferation of the generated lines in mixed lymphocyte cultures was significantly reduced when compared to unmanipulated PBMCs [66].

The above method of generating ADV-specific T cells was adopted clinically in 2006 by Feuchtinger's group for nine children with systemic ADV infection after HSCT for mainly leukaemia or lymphoma. These children underwent myeloablative conditioning regimen with T-cell depletion for HSCT and had ADV viraemia not controlled by antivirals. T-cell transfer was performed if a sufficient ADV-specific T-cell response was detected in the donor (>0.01% of T cells). A mean of 14 × 10^3^/kg (range 1.2–50 × 10^3^/kg) T cells were infused at a median of +77 days after transplant (range +40–+378). T-cell infusion was well tolerated in all nine patients, except for one case with aggravation of preexisting skin GVHD that was seen at days 10 to 14. Five out of six evaluable patients had significant decrease in viral DNA in peripheral blood and stool with an *in vivo *expansion of specific T cells. Those without a specific T-cell response after adoptive T-cell transfer had either increasing or unchanged viral DNA load in peripheral blood. Three patients in whom followup was possible had sustained ADV-specific T-cell response detected 4 to 6 months after T-cell transfer. Efficacy was independent of T-cell dose transferred, suggesting efficient *in vivo *expansion. Four patients died of whom 3 died from adenovirus-associated, preexisting multiorgan failure. Three out of 4 patients who died did not have specific T-cell response after immunotherapy [67].

Chatziandreou et al. also reported on the successful isolation of ADV-specific T cells using a similar protocol [69]. Using the Miltenyi IFN-*γ* secretion and capture assay with adenovirus lysate, ADV-specific T cells were isolated, expanded, and restimulated over 2 weeks. The numbers of eluted virus-specific cells from six ADV-positive donors ranged from 1 to 7 × 10^5^ cells, with the majority being CD4+ cells. After a 2-week culture period, a 1.5 to 2 log expansion was seen with cell numbers averaging at 1 × 10^7^ cells. This would enable infusions of up to 10^5^ ADV-specific cells/kg for most adults and larger amounts of paediatric patients. This approach offers the advantage of a short 14-day culture period, allowing for generation of cells in response to first detection of virus during routine screening. It is therefore less labour intensive and has a more favourable cost: benefit profile [69]. Using a similar IFN-*γ* capture protocol, five patients have been treated at Great Ormond Street Hospital with ADV-specific T cells either from the original donor (*n* = 3) or third-party haploidentical parents (*n* = 2) [60]. All 5 children had undergone either *in vivo *or *ex vivo *T cell depletion as part of their conditioning regimen and had peak ADV loads in blood ranging from 5.6 × 10^4^/mL to 22 × 10^6^/mL before cell infusion. IFN-*γ* secreting ADV-specific T cells in the donations were enriched to between 19 and 64% after 24 hours and infused directly without *ex vivo* expansion (Figure 3(a)), with 4 children receiving 10^4^ T cells/kg and 1 child receiving 10^5^ T cells/kg at an average of 80 days after the original stem cell graft. Three patients cleared ADV in blood after a single infusion of 10^4^/kg and had demonstrable ADV-specific T cells in circulation detected by IFN-*γ* secretion assay. No acute, infusion-related toxicities were observed. Three patients died: one due to bystander GVHD after cell infusion even though viraemia had resolved [70], the other two failed to clear virus and died at days 175 and 56, respectively [60]. 

## 10. Peptide Expanded T Cells

More recently in order to generate CTLs from a greater majority of healthy donors in a short period of time, Comoli et al. used a pool of five 30 mer peptides derived from HAdV5 hexon protein, to generate 21 T-cell lines with limited alloreactivity starting from median of 20 × 10^6^ donor PMBC and expanded to 75 × 10^6^ cells at the end of 26 days. This would have been sufficient for infusion aimed at 0.5 × 10^6^ cells/kg [71]. In 2010 Aïssi-Rothe et al. used clinical-grade PepTivator-ADV5 Hexon (Miltenyi Biotec, Germany) and 6 hr incubation time to generate IFN-*γ* secreting ADV-specific T cells which were expanded over a median of 2-week period with IL2 and irradiated autologous feeder cells (Figure 3(b)). Up to 85 × 10^6^ ADV T cells were generated with a mean of 1.7 log expansion and a reduction of 1.3 log in alloreactivity [61].

## 11. Stimulation with Viral DNA Plasmids

In 2011 the Baylor group took an alternative approach to rapidly select virus-specific T cells. Instead of using adenovectors to stimulate T cells, dendritic cells nucleofected with DNA plasmids encoding LMP2, EBNA1, and BZLF1 (EBV), hexon and penton (ADV), and pp65 and IE1 (CMV) were used as antigen-presenting cells. Secondly, EBV-LCLs were removed and replaced by gas permeable culture device (G-Rex) that promotes expansion and survival of large cell numbers after a single stimulation. Activated T-cells were cultured in the presence of IL-4 (1,000 u/mL) and IL-7 (10 ng/mL). This approach reduced the time of manufacturing from 10 weeks to 10 days, as well as the cost of production by >90% [72]. Using this method, 22 trivirus and 14 bivirus CTL lines were produced with a 1.5 log expansion from 15 × 10^6^ starting PBMCs. 10 patients with viral reactivation (either single or dual) were treated between day 27 and 52 months after HSCT, with each patient receiving 0.5 to 2 × 10^7^ cells/m^2^. Complete virological responses associated with increased frequency of virus specific T cells were seen in 80%. One patient developed stage 2 skin GVHD after infusion but no other toxicities were observed [68]. Similarly this approach was used to develop a single preparation of polyclonal (CD4+ and CD8+) CTLs that is specific for 7 viruses (EBV, CMV, adenovirus, BK, human herpes virus 6, respiratory syncytial virus, and influenza) [73].

## 12. Isolation Protocols Using T-Cell Activation Markers

Apart from using IFN-*γ* production as a way to capture antigen-specific T cells, alternative isolation strategies based on other T-cell activation markers have been investigated. Khanna et al. generated antigen-specific T cells lines for ADV, EBV, CMV, A fumigatus, and C albicans based on magnetic cell separation of CD154+ T cells after 16 hours of stimulation with antigens, followed by expansion in presence of IL2, IL7, and IL15 over 14 days. Purity of the product was between 8 to 15%, with a higher frequency of virus-specific T cells compared to fungus-specific T cells [74]. Leibold et al. compared the specificity, expansion/differentiation potential, and Th1 response against CMV and ADV after isolation of antigen specific T cells based on IFN-*γ* release or expression of activation markers (CD137 ND CD154). Isolation of T cells based on expression markers is feasible and less time consuming, but it resulted in smaller proportion of Th1 cells compared to IFN-*γ* capture which may correspond to less effector function *in vivo* [75]. 

Because CD4+ T cells are critical in human ADV infection, Haveman et al. explored the possibility to selectively expand and isolate ADV-specific CD4+ T cells. PBMCs were stimulated with 15 mer pan-DR binding CD4+ T cell epitopes of ADV serotype 5 peptides using artificial APCs, composed of liposomes harbouring ADV peptide/HLA class-II complexes [76]. The resultant T-cell lines after 7-day culture period produced mainly proinflammatory cytokines (TNF-*α*, IFN-*γ*, MDC, RANTES, and MIP-1*α*), expressed perforin and granzyme B, had specific ADV-killing, and were not alloreactive [76].

Table 2 summarises recent clinical trials on the use of virus-specific T cells.

## 13. Financial Implications

The economic burden of viral reactivation has been assessed recently at one of the main Paediatric transplant centres in the UK. By calculating the cost of antiviral drugs and excess inpatient hospital stay, viral reactivation costs an estimate of *£*22500 per patient (compared to *£*800 per day for routine inpatient costs following HSCT) [36]. On the other hand, although generating virus-specific T cells can be a costly operation, it could result in less patients with ADV infection requiring prolonged hospital stay and/or ICU admissions. Advances are being made in cell production techniques to reduce production time and generation of single CTL product with specificity against multiple viruses will be more cost effective. 

## 14. Summary

It is undeniable that adenovirus can cause significant morbidity and mortality in immunocompromised children. Current antiviral therapy with cidofovir is not always successful, although current available data on the new drug CMX001 seems promising. Ultimately clearance of adenovirus requires reconstitution of T-cell immunity which is often delayed after haematopoietic stem cell transplant, especially in T-cell depleted grafts. Major advances have been made over the past decade in adoptive transfer of virus-specific T cells. However there is still ground to be covered to move T-cell immunotherapy from specialist centres to standard-of-care therapy available to all transplant recipients. 

## Figures and Tables

**Table 1:** Classification of human adenoviruses and their sites of infection.

Subgroup	Serotype	Sites of infection
A	12, 18, 31	Gastrointestinal

B1	3, 7, 16, 21, 50	Respiratory

B2	11, 14, 34, 35	Urinary tract/renal

C	1, 2, 5, 6	Respiratory

D	8, 9, 10, 13, 15, 17, 19, 20, 22–30, 32, 33, 36, 37, 38, 39, 42–48, 49, 51	Eye

E	4	Respiratory

F	40, 41	Gastrointestinal

**Table 2:** Clinical trials using virus-specific cytotoxic T cells in the HSCT setting.

Reference number (centre)	Virus specificity	Expansion protocol	Antigen used	Infused number and type of cells	Patients treated	Clinical results
[62] (Texas)	EBV, CMV, ADV	Donor PBMCs infected with vector and restimulated, repetitively, with irradiated EBV-LCLs transduced with same vector over 10–12 weeks	Clinical-grade Ad5f35pp65 vector	Median 5 × 10^7^ polyclonal cells/m^2 ^infused at 35–150 d after HSCT (median 62 d)	11 infused (children and adults; 10 prophylactically, 1 treated for ADV infection)	3/3 cleared CMV and 3/3 cleared EBV infection/PTLD without antivirals; 3 patients with infection and 1 with disease cleared ADV after-CTL. No GVHD

[67] (Tuebingen)	ADV	IFN-*γ* selection after 16 h stimulation Cytokine-secreting cells magnetically enriched	Adenoviral antigen type C (nonclinical grade)	1.2–50 × 10^3^/kg ADV-reactive polyclonal T cells infused	9 children with ADV infection	5 out of 6 with ADV responded 1 died at 30 days from ADV infection 5 deaths (3 due to ADV infection)

[64] (Texas)	EBV + ADV	PBMCs infected with vector Responder cells restimulated weekly with irradiated autologous LCL transduced with the same vector IL-2 being added twice weekly from day 14. CTLs cryopreserved after 3 or 4 simulations	Ad5f35^null^ vector MOI 200 vp/cell	20 CTL lines with EBV and ADV specificity produced, 13 lines infused Dose of 5 × 10^6^ to 1.35 × 10^8^ cells/m^2^ at 40 to 150 days after HSCT (median 77 days)	13 children [(M)MUD or haplo] 2 with active ADV disease; 11 prophylactic	No toxicities or GVHD, monitored for 3 months. Only detected increases in ADV-sp T cells in peripheral blood in those with active ADV infection (2 out of 13)

[60] (London)	ADV	PBMCs stimulated for 16 hrs with ADV-hexon antigen Cytokine-secreting cells selected using anti-IFN-*γ*microbeads and Miltenyi Mini-MACS column within 24 h	Commercially available purified ADV-hexon antigen (Binding Site, UK)	3 received *γ*-captured cells from original stem cell donor Cells (10^4^–10^5^/kg) received on average 80 d after original graft (range 34–122)	5 patients treated (3 with original donor; 2 third-party haploidentical donor)	Blood viraemia resolved in 3 IFN-*γ* secreting ADV-specific T cells present in 4 patients 3 died—1 of bystander GVHD after clearing virus

[65] (Texas)	ADV, CMV, EBV	Banked 3rd party PMBCs transduced with vector and stimulated with EBV-LCL transduced with same vector	Ad5f35pp65 vector	32 virus-specific lines from individuals with common HLA polymorphisms immune to EBV, CMV, or ADV Each patient received up to 2 × 10^7^ cells/m^2^	18 lines administered to 50 patients with severe viral illness with one of the viruses	Cumulative rates of complete or partial responses at 6 weeks were 74% for the whole group 2 de novo GVHD (grade 1).

[68] (Texas)	ADV, CMV, EBV	Donor PBMCs stimulated with nucleofacted DCs and cultured over 2-3 weeks with IL4 and IL7.	DCs nucleofacted with range of EBV, CMV, and ADV viral antigens	22 trivirus and 14 bivirus CTL lines. Each patient received 0.5–2 × 10^7^ cells/m^2^	10 patients with viral reactivation treated between day 27 and month 52 after HSCT	Viral clearance and increased frequency of VSTs in 80% 1 stage 2 skin GVHD
